# Cutaneous manifestations of Fabry disease: A systematic review

**DOI:** 10.1111/1346-8138.17690

**Published:** 2025-03-07

**Authors:** Rami Nabil Al‐Chaer, Mathias Folkmann, Nina Løth Mårtensson, Ulla Feldt‐Rasmussen, Mette Mogensen

**Affiliations:** ^1^ Department of Dermatology, Bispebjerg and Frederiksberg Hospital Copenhagen University Hospitals Copenhagen Denmark; ^2^ Department of Nephrology and Endocrinology Copenhagen University Hospitals Rigshospitalet Copenhagen Denmark; ^3^ Department of Pathology Copenhagen University Hospitals Rigshospitalet Copenhagen Denmark; ^4^ Department of Clinical Medicine, Faculty of Health and Medical Sciences University of Copenhagen Copenhagen Denmark

**Keywords:** Anderson–Fabry, angiokeratoma, cutaneous manifestations, hypohidrosis, lymphoedema, telangiectasia

## Abstract

Fabry disease (FD) is a rare X‐linked lysosomal storage disorder resulting in potential debilitating accumulation of glycosphingolipids in organs such as skin, nerves, heart, kidneys, lungs, and the central nervous system. Skin is easily investigated and can guide clinicians to diagnose FD, minimizing delay of enzyme substitution therapy. This systematic review followed the PICO and PRISMA guidelines. Using Web of Science, PubMed, and Embase, a total of 968 studies were retrieved by January 1, 2024. All clinical studies describing the skin characteristics and abnormalities of FD patients were included. After inclusion of articles, the methodological quality was assessed using the QUADAS‐2 critical appraisal checklist. Twenty‐three studies were included. Different skin manifestations were described in FD patients. Fifteen studies described angiokeratomas, five studied telangiectasias, 13 studied sweat abnormalities (anhidrosis/hypohidrosis/hyperhidrosis), nine described lymphoedema, and two reported hair abnormalities. Sweat abnormalities were the most common skin manifestation, affecting 57.6% of patients with FD; angiokeratomas were observed in 51.5% of patients. A high prevalence (16.5%) of lymphoedema was seen in a large study (*n* = 5487). Skin involvement appeared age‐dependent and increased with age. Quality assessment showed high or unclear risk of bias in 19/23 studies. We summarized data on skin manifestations in 10 757 FD patients. The pathogenesis of sweat abnormalities and the occurrence of cutaneous vascular lesions, such as angiokeratomas and telangiectasias, in only half of FD patients remains poorly understood. Enzyme replacement therapy generally did not reduce skin manifestations in FD patients. Direct comparisons between studies were challenging due to variations in reported outcomes.

## 1 INTRODUCTION

Fabry disease (FD), also referred to as Anderson–Fabry disease, can be detrimental to normal development in affected children and early diagnosis is imperative for proper management of families with variants of the *GLA* gene.^1^ Lysosomal storage diseases are a group of more than 45 distinct inherited metabolic disorders caused mostly by enzyme deficiencies within the lysosome resulting in progressive accumulation of undegraded substrate and leading to a broad spectrum of clinical manifestations.^2^ FD is one of them,^3^, ^4^, ^5^ an X‐linked disorder caused by a deficiency of the lysosomal enzyme α‐galactosidase A. The deficiency causes a progressive accumulation of glycosphingolipids, primarily globotriaosylceramide (Gb3).^6^ This accumulation can occur in single or multiple organs, resulting in progressive cellular dysfunction, development of fibrosis, and ultimately organ damage and failure.^7^

In FD, the *GLA* genotype, characterized by genetic variations in the *GLA* gene, gives rise to a diversity of clinical phenotypes, encompassing a spectrum of clinical manifestations that affect multiple organ systems, with variable onset, severity, and progression. *GLA* variants are classified as classic, late‐onset variants of unknown significance or benign polymorphism. FD clinical phenotypes are classified as either classic or late‐onset.

**FIGURE 1:**
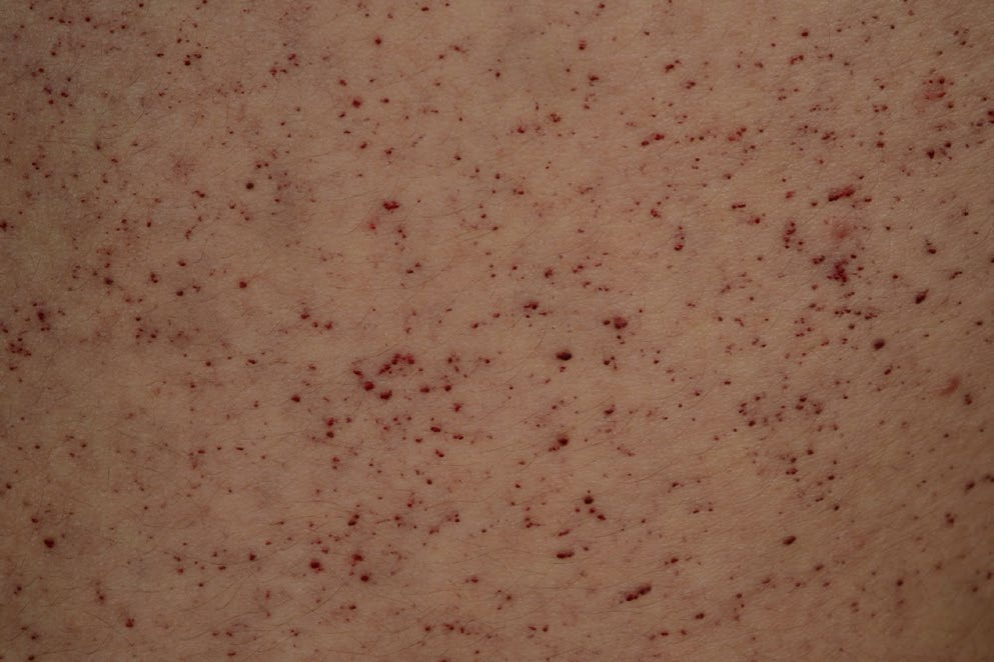
Clinical photograph of multiple angiokeratomas on the trunk of a young patient with Fabry disease. Some of the angiokeratomas are large and deep‐seated, more telangiectatic. This photograph illustrates why various vascular lesions are referred to as cutaneous vascular lesions in this review. Courtesy of M.M., shown with permission from the patient.

Involvement of the skin is very common in FD, and the skin involvement was the organ that led to discovery and description of the disease by two independent dermatologists in 1898.^4^, ^5^ The characteristic cutaneous hallmark lesions in FD patients are superficial angiomas and angiokeratomas.^8^ Other cutaneous, adnexal, and connective tissue manifestations include common telangiectasias, peripheral oedema, lymphoedema, hypohidrosis, and scant body hair.^9^ Early manifestations of FD can sometimes be seen in childhood and are mostly reported as angiokeratomas, hypohidrosis, and gastrointestinal symptoms with stomach pain, diarrhea, and borborygmi. Skin manifestations as well as characteristic corneal opacities usually appear between the ages of 5 and 13 years but may occur earlier during infancy.^9^ More serious complications include heart failure, ischemic stroke, Fabry nephropathy with proteinuria, progressive kidney failure, and hearing loss.^7^, ^10^ The number of skin lesions increases with age in most patients.^7^, ^10^ Angiokeratomas typically appear as clusters of deep red dots consisting of convoluted blood vessels in the skin (Figure 1). Angiokeratomas are elicited by accumulation of undegraded glycosphingolipids, especially in vascular endothelium.^11^, ^12^ Similar angiomas commonly appear with age in individuals not affected by Fabry, referred to as “cherry spots”. Angiokeratoma lesions in FD patients typically appear on the trunk, genitals, thighs, and face but can also be detected in other parts of the body.^9^, ^13^ Nonetheless, several cases of FD patients without angiokeratomas have been reported.^14^, ^15^ The pathogenesis of hypohidrosis and anhidrosis in FD is still not fully understood. It may be a result of Gb3 accumulation in sweat glands and/or in their respective vascular and neuronal networks.^12^, ^16^, ^17^ As a result, it may impair a patient's tolerance to heat and exercise.^18^

This systematic review aimed to investigate the existing literature on skin manifestations associated with FD with the following three purposes: (i) to provide an overview of skin disorders associated with FD that have been examined either clinically and/or by diagnostic imaging methods; (ii) to analyze quantitative data regarding the extent, localization, severity, and other relevant features in skin manifestations; and (iii) to provide recommendations for the early diagnosis and treatment of the skin manifestations in research and clinical work.

## 2 MATERIALS AND METHODS

### 2.1 Project registration

This systematic review focused on the skin manifestations associated with FD and is reported in accordance with the PRISMA guidelines.^19^ Prior to the start, the review protocol was submitted to the International Prospective Register of Systematic Reviews (PROSPERO) on December 14, 2023 and was officially registered on December 23, 2023 with the code CRD42023492251.

### 2.2 Study criteria

Studies included reported any clinical assessment of skin changes or skin manifestations in FD patients, such as angiokeratomas, telangiectasia, hair growth, hypohidrosis, and other skin changes described in patients with FD evaluated by the naked eye or by well‐known diagnostic imaging technologies. The results contained details about the type of skin change/disease, localization, severity, and symptoms. These were compared with the reference standard of normal skin, including age‐related changes in healthy individuals. Clinical studies using in vivo diagnostics and imaging including studies describing changes before and after treatment were included, therefore peer‐reviewed reports and original papers, including case reports with more than five FD patients were also included. Histological and other ex vivo outcomes were excluded. Studies in languages other than English, Danish, Norwegian, or Swedish were not included. Animal, laboratory, gene therapy, and quality of life studies, including in vitro trials, were excluded. Reviews, guidelines, and involvement of other organs or drug therapy as main topics were also excluded.

### 2.3 Search strategy

**FIGURE 2:**
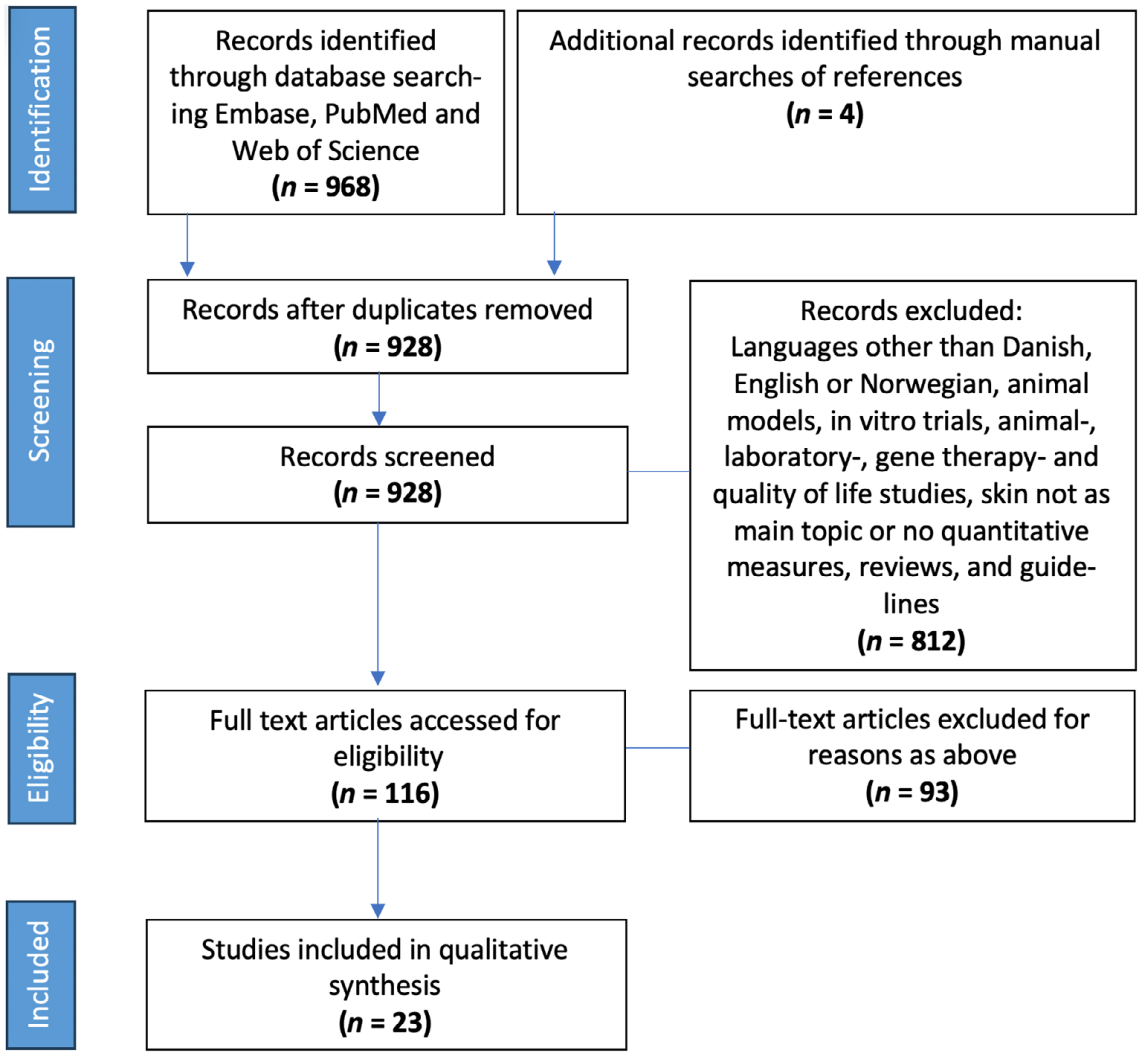
An electron microscopy histology image of a kidney from a typical Fabry patient showing intracytoplasmatic lipid inclusions, with the characterized lamellated/striped “zebra bodies” in the podocytes (yellow arrow), which are the most affected cell affected in Fabry's disease.

On January 1, 2024, a final systematic search was performed in the PubMed, Web of Science, and Embase databases using different MeSH terms to retrieve studies on FD and skin manifestations. To also include literature to which MeSH terms were not attached, words in the text were also used in the literature search. Reference lists were manually searched for additional studies to include. The search was performed according to PRISMA guidelines.^19^ As skin manifestations in FD patients are wide, various search terms involving different skin manifestations were used to ensure the search was systematic and wide. The following search string was used ((“fabry”[All Fields] OR “fabry's”[All Fields] OR “angiokeratoma corporis diffusum”[All Fields]) AND (“skin”[All Fields] OR “lymphedema”[All Fields] OR “angiokeratoma”[All Fields] OR “angiokeratomas”[All Fields] OR “hemangioma”[All Fields] OR “angioma”[All Fields] OR “angiomas”[All Fields] OR “hypohidrosis”[All Fields] OR “hyperhidrosis”[All Fields] OR “anhidrosis”[All Fields] OR “sweat”[All Fields] OR “sweating”[All Fields] OR “hair”[All Fields] OR “telangiectasiae”[All Fields] OR “telangiectasia”[All Fields] OR “telangiectasias”[All Fields])). Figure 2 illustrates the search profile.

### 2.4 Study selection

The literature search was done by authors R.N.A.C. and M.M., both of whom screened all titles and abstracts to identify studies meeting the described study criteria. Authors were not blinded regarding the authors and affiliation names of these studies. Full‐text articles of potentially relevant studies were obtained and assessed. Any disagreement was solved by consensus.

### 2.5 Data extraction

The articles were read, and one author (RNAC) extracted the following information from the included studies and registered it in a spreadsheet (Microsoft Excel 2021): first author, year of publication, and study design including age, number, and sex of patients. Furthermore, details about the index test (clinical and/or imaging technique), reference standard, and details about the type of skin change/disease, anatomical localization, severity, symptoms, and histopathological results were also extracted.

### 2.6 Quality assessment

A risk of bias assessment was performed for all included articles using the QUADAS‐2 critical appraisal checklist.^20^, ^21^ QUADAS‐2 is recommended for use in systematic reviews to assess the risk of bias and the applicability of primary diagnostic accuracy studies. It consists of four key domains: patient selection, index test, reference standard, and flow and timing. The studies were scored “low”, “high,” or “unclear”. Studies that scored “low” in all four domains were judged “low risk of bias and low concern regarding applicability”. If a study was judged “high” or “unclear” in one domain, it was judged “at risk of bias” or “concerns regarding applicability”, respectively.

### 2.7 Data analysis and synthesis

Data analysis with descriptive statistics, such as mean values, percentages, ratios, and confidence intervals, was performed. If data were unavailable, this was explicitly stated. Finally, due to lack of quantitative data, a narrative data synthesis was carried out for all included articles.

## 3 RESULTS

### 3.1 Literature search

A total of 968 records were identified across the databases of Embase, Web of Science, and PubMed (Figure 4).

### 3.2 Study characteristics

The 23 included papers consist of one randomized controlled trial (RCT), one prospective, controlled, non‐randomized clinical study, four retrospective studies, eight cross‐sectional and nine case series (*n* = 10.757 patients in total [controls included], *n* = 2396 patients with any skin manifestation) describing skin manifestations in FD.

Fifteen of the 23 included studies described angiokeratomas,^22^, ^23^, ^24^, ^25^, ^26^, ^27^, ^28^, ^29^, ^30^, ^31^, ^32^, ^33^, ^34^, ^35^, ^36^ five studied telangiectasia,^22^, ^30^, ^32^, ^36^, ^37^ 13 described sweat abnormalities (anhidrosis/hypohidrosis/hyperhidrosis),^22^, ^23^, ^24^, ^25^, ^26^, ^27^, ^29^, ^30^, ^31^, ^33^, ^36^, ^38^, ^39^ nine described lymphoedema,^22^, ^26^, ^27^, ^30^, ^34^, ^37^, ^40^, ^41^, ^42^ and two described hair abnormalities.^25^, ^43^ The characteristics of the included studies are summarized in Table 1.

**TABLE 1:** Study characteristics.

References	Study type	No. of patients (F, females; M, males)	Mean age, years (mean ± SD)	Study methodology	Conclusion and highlights of studies
Alkhatib et al. (2023)^40^	Retrospective study	7671 (F = 4308, M = 3363)	F = 52, M = 44	Data extraction	Lymphoedema is a common manifestation of FD, occurring in 16.5% of the patients assessed for lymphoedema, with a tendency to present later in females Classic phenotype has the highest prevalence of lymphoedema
Amann‐Vesti et al. (2003)^42^	Case series	22 (F = 11, M = 11)	F = 38.2, M = 46	Fluorescence micro‐lymphography, lymph capillary pressure, physical examination, duplex sonography	High variability of the diameter of the lymphatics is seen in FD patients
Anker et al. (2023)^22^	Cross‐sectional	26 (F = 14, M = 12)	F = 42.1 ± 19.9, M = 31.1 ± 21.7	Non‐linear optical microscopy, multispectral imaging, dermoscopy, physical examination	Angiokeratomas was the most common manifestation and could be morphologically distinguished by non‐linear optical microscopy Clinical heterogeneity of FD can result in a diagnostic delay
Eng et al. (2007)^44^	Retrospective study	1765 (F = 812, M = 953)	F = 32, M = 23	Data extraction	In females, diagnosis is typically delayed by 19 years, while in males, the delay averages 14 years.
Galanos et al. (2002)^23^	Cross‐sectional study	67 (F = 38, M = 29)	n/a	Physical examination	Findings were variable between patients In females, anhidrosis predicted the presence of significant FD renal disease Females are often symptomatic, and early diagnosis and treatment might be beneficial
Groot et al. (1968)^43^	Case series	29 (F = n/a, M = n/a)	n/a	Physical examination	Findings were variable, with patients showing no angiokeratomas to a great many Tiny dilatations of the scrotal vessels and peculiar beard growth were present Clinical manifestations in FD are extremely variable
Guinovart et al. (2013)^24^	Case series	5 (F = 1, M = 4)	F = 36, M = 29.25 ± 15.50	Physical examination	Patients had angiokeratomas, ranging from a typical bathing trunk area appearance to an atypical distribution, with lesions on the palms, mouth, umbilicus, vulva, and trunk ERT did not improve any of the lesions Angiokeratomas should prompt the dermatologist to consider FD diagnosis Young relatives of newly diagnosed patients should also be evaluated
Gupta et al. (2008)^38^	Prospective, controlled, non‐randomized clinical study	49 (F = 0, M = 49)	n/a	Dynamic dermal impedance monitor	All patients have hypohidrosis No difference was found between patients on ERT and those naive to ERT Dynamic dermal impedance monitor is useful as a clinical tool in assessing differences in skin moisture
Larralde et al. (2004)^25^	Case series	11 (F = 5, M = 6)	F = 49.4, M = 23	Electron microscopy, physical examination, skin biopsy	Older males had more angiokeratomas (>100) with morphologic characteristics such as punctate angiectases 1‐ to 10‐mm red to black papules and plaques with verrucous surface Angiokeratomas and hypohidrosis can be early diagnostic clues to FD
Lidove et al. (2016)^37^	Retrospective study	40 (F = 25, M = 15)	F = 44.2, M = 40.1	Physical examination	Lymphedema and telangiectasia were reported in combination with musculoskeletal manifestations FD diagnosis is usually delayed due to confusion with common disorders
MacDermot et al. (2001a)^27^	Cross‐sectional study	98 (M = 98, F = 0)	M = 34.8	FD specific questionnaire	Males had multiple disease manifestation, including skin manifestations such as angiokeratomas, hypohidrosis and lymphedema The mean age of onset for angiokeratomas was found to be 16.8 years
MacDermot et al. (2001b)^26^	Cross‐sectional study	60 (M = 0, F = 60)	F = 44.9	FD‐specific questionnaire	Females had multiple disease manifestations, including skin manifestations such as angiokeratomas, hypohidrosis and lymphedema
Wataya‐Kaneda et al. (2023)^28^	Case series	4 (F = 1, M = 3)	F = 47, M = 20.6	Physical examination, evaluation of treatment with topical 0.2% sirolimus gel	In one patient, seven out of nine angiokeratomas did not respond to sirolimus treatment. Sirolimus gel was as effective as systemic sirolimus treatment in patients with venous and capillary malformations, and for early active lesions, including systemic venous malformations
Möhrenschlager et al. (2007)^45^	Cross‐sectional study	31 (F = 19, M = 12)	F = n/a, M = n/a	Questionnaire, physical examination, serum IgE concentration	FD patients have increased serum IgE concentrations and show symptoms of atopic disorders in a prevalence comparable to healthy individuals
Møller et al. (2009)^39^	Cross‐sectional	38 (F = 38, M = 0)	F = 44.8	Quantitative Sudomotor Axon Reflex Test	FD patients had a reduced sweat output to iontophoresis of acetylcholine Sweat output was significantly different for those with painful neuropathy compared to healthy controls, but not for the non‐pain group Sweat output decreased significantly with increasing age in FD patients
Nagai‐Sangawa et al. (2021)^29^	Case series	17 (F = 1, M = 16)	F = 30, M = 33.5 ± 11.42	Electron microscopy, physical examination, skin biopsy, thermoregulatory sweat test (iodine‐starch method)	FD should be suspected in patients with hypohidrosis
Orteu et al. (2007)^30^	Retrospective study	714 (F = 369, M = 345)	F = 35.1 ± 15, M = 38 ± 18.1	Data extraction	All patients were actively treated with ERT 78% of males and 50% of females had one or more skin manifestations, the most common being angiokeratomas The presence of dermatological manifestations such as angiokeratomas and telangiectasia appears to be a marker of greater disease severity as judged by the MSSI
Spence et al. (1978)^31^	Case series	18 (F = 0, M = 18)	M = 24 ± 10.88	Physical examination, questionnaire about symptoms	The degree of hypohidrosis did not correlate with the frequency or severity of Fabry crises Variability in symptoms such as angiokeratomas and sweat abnormalities suggests that genetic and environmental factors play a significant role in the patient clinical outcome
Wallace et al. (1965)^32^	Case series	5 (F = 1, M = 4)	F = 55, M = 27.75 ± 2.68	Physical examination, skin biopsy	Angiokeratomas were described as reddish blue to black raised spots 1–5 mm in size on the bathing trunk area and upper extremities FD was proven in three males and remained a possibility in two cases
Wattanasirichaigoon et al. (2006)^33^	Case series	17 (F = n/a, M = n/a)	n/a	Physical examination	All patients had the mutation L106R No patients had angiokeratomas Two male patients had hypohidrosis and one had hyperhidrosis Females had no sweat abnormalities Physical examination may not provide clues for diagnosis, especially if typical skin lesions are absent
Whybra et al. (2001)^34^	Cross‐sectional study	20 (F = 20, M = 0)	F = 38	Physical examination	Angiokeratomas and lymphedema were present in 11 and 10 patients, respectively. Lymphoedema was found in the lower legs, feet, and wrists ERT in females who do not yet have severe symptoms should be considered
Wijburg et al. (2015)^35^	RCT, open‐label	31 (F = 0, M = 31)	M = 12	Light microscopy, physical examination, skin biopsy	Gb3 accumulation was documented in skin capillary endothelial cells 23 of 29 patients had mild or moderately abnormal Gb3 accumulations in deep vessel endothelial cells ERT should be considered early on as it might improve long‐term outcome
Zampetti et al. (2013)^36^	Cross‐sectional	35 (F = 16, M = 19)	43	ELISA, physical examination, serum VEGF‐A measurement	Mean serum concentration of VEGF‐A in the FD group was significantly increased A significant association between VEGF‐A levels and skin manifestations, including angiokeratomas, and sweating abnormalities was found VEGF‐A levels were increased in patients, possibly due to vascular damage characterizing FD

Abbreviations: ELISA, enzyme‐linked immunosorbent assay; ERT, enzyme replacement therapy; FD, Fabry disease; Gb3, globotriaosylceramide; IgE, immunoglobulin E; MSSI, Mainz Severity Score Index; n/a; not applicable; RCT, randomized controlled trial; SD, standard deviation; VEGF‐A, Vascular Endothelial Growth Factor A.

### 3.3 Patient characteristics

Findings are summarized in Tables 1 and 2. Sweat abnormalities were the most common skin manifestation, affecting 57.6% of patients (95% confidence interval [CI] 54.7%–60.5%; Table 2). Angiokeratomas were observed in 51.5% of patients (95% CI 48.3%–54.2%), while telangiectasia, lymphoedema, and hair abnormalities affected 19.9% (95% CI 17.1%–22.7%), 15.7% (95% CI 14.9%–16.6%), and 7.5% (95% CI 0.1%–15.7%) of patients, respectively.

**TABLE 2:** Skin manifestations in FD patients: the number of patients with angiokeratomas, telangiectasia, sweat abnormalities, lymphoedema, and hair abnormalities compared to all examined Fabry patients.

	Angiokeratomas	Telangiectasia	Sweat abnormalities	Lymphedema	Hair abnormalities
Number of patients with skin manifestations	578	156	639	1020	3
Number of patients examined for skin manifestations	1128	785	1109	6486	40
Percentage with changes	51.5%	19.9%	57.6%	15.7%	7.5%
95% CI	0.48, 0.54	0.17, 0.23	0.547, 0.61	0.149, 0.166	0.001, 0.16

Abbreviation: 95% CI, 95% confidence interval; FD, Fabry disease.

A wide range of different variables were measured in each study. As a result, the data are presented in the form of a narrative systematic review, but with a table presenting quantitative analysis.

### 3.4 Skin manifestations

Angiokeratomas were the second most common manifestation, mentioned in 14 studies (eight case series, five cross‐sectional studies, one retrospective study, one RCT, *n* = 1.112 patients).^22^, ^23^, ^24^, ^25^, ^26^, ^27^, ^28^, ^29^, ^30^, ^31^, ^32^, ^33^, ^34^, ^35^, ^36^ Telangiectasia was the third most common manifestation, mentioned in five studies (two retrospective studies, two cross‐sectional studies, one randomized controlled trial, one case series, *n* = 785 patients).^22^, ^30^, ^32^, ^36^, ^37^

In a large cohort, 78% of males and 50% of females had one or more skin manifestations, the most common being angiokeratomas (66% males, 36% females), hypohidrosis (53% males, 28% females), telangiectasia (23% males, 9% females), and lymphoedema (16% males, 6% females).^30^ The presence of angiokeratomas and telangiectasias correlated with the severity of the systemic manifestations of the disease, as assessed by the Mainz Severity Score Index (MSSI).^30^ All patients were in active treatment with enzyme replacement therapy (ERT). One study found a median age of onset of skin manifestation in females and males at 17 and 9 years and percentages with skin manifestations of 12% and 31%, respectively.^44^ Another study found that the mean age of onset of angiokeratomas in males was 16.8 years.^27^

Gb3 accumulation was documented in superficial skin capillary and deep vessel endothelial cells of 23 of 29 patients who had mild or moderately abnormal Gb3 accumulations in deep vessel endothelial cells.^35^

Nagai‐Sangawa et al. described a patient with hypohidrosis who had few erythematous‐purple papules on the trunk and extremities.^29^ Ultrastructural examination of the papules revealed angiokeratomas with lamellar inclusions in endothelial cells.^29^ In five male patients, angiokeratomas affected the genitalia, back, elbows, and other frequently traumatized areas.^25^ All patients suffered from hypohidrosis.^25^ Older males exhibited more angiokeratomas (>100) with morphological characteristics, such as punctate angiectasias, 1‐to 10‐mm red to black papules, and plaques with a verrucous surface.^25^ Electron microscopy of skin biopsies revealed stromal cells in male tissue, and endothelial and smooth muscle cells in females containing membrane‐bound inclusions with a lamellar structure.^25^ Figure 2 illustrates a representative electron micrograph of a kidney biopsy from a FD patient.

Angiokeratoma skin histology showed dilated capillary spaces in the upper portion of the dermis and the epidermis. A special stain, Sudan black B, did not show demonstrate any lipid within the walls of the vessels^32^ and the lesions in Fabry patients could not be distinguished from angiokeratomas in other patients illustrated in Figure 4. Ectatic capillaries in the papillary dermis, epidermal acanthosis, most prominent at the edges of the lesion, and hyperkeratosis have previously been described.^30^

Angiokeratomas and hemangiomas visualized by ex vivo nonlinear microscopy showed a hyperkeratotic surface and ectatic dilated capillaries with acanthosis limited to the papillary dermis in angiokeratomas.^22^ In hemangiomas, thin capillaries divided by thin fibrous collagen septae extended further towards the subcutaneous tissue, suggesting a proliferative process.^22^ Hemangiomas had multiple vascular spaces and normal epitelium.^22^

Guinovart et al. reported various angiokeratomas in a case series of five patients, ranging from a typical bathing trunk area appearance to an atypical distribution, with lesions of the palms, mouth, umbilicus, vulva, and trunk.^24^ A patient had extensive angiokeratomas with a left‐sided distribution.^24^ Anker et al. found angiokeratomas in 24 out of 26 patients.^22^ Dermoscopy of angiokeratomas showed lacunae and dotted vessels, with a whitish veil.^22^ Autofluorescence (405 nm) and diffuse reflectance (526 nm) images showed the underlying vasculature more prominently compared to dermoscopy.^22^ In non‐linear optical microscopy, angiokeratomas and hemangiomas could be morphologically differentiated.^22^

One study described angiokeratomas as small reddish blue to black raised spots 1–5 mm in diameter located in the bathing trunk area and upper extremities.^32^ A patient with angiokeratomas was treated with topical sirolimus, but the treatment was effective in only two out of nine lesions.^32^

A case series of 17 patients with the L106R mutation in the GLA gene^33^ found no instances of angiokeratomas. Another study reported variable clinical manifestations in FD patients, with some showing minimal or no angiokeratomas, while others exhibited a substantial number.^43^ Additionally, two studies concluded that skin manifestations did not improve with ERT.^24^, ^38^

Sweat abnormalities, particularly hypohidrosis, were the second most investigated manifestations, mentioned in 12 studies (one prospective, controlled, non‐randomized clinical study, one retrospective study, five cross‐sectional studies, four case series, *n* = 1.109 patients).^22^, ^23^, ^24^, ^25^, ^26^, ^27^, ^29^, ^30^, ^31^, ^33^, ^36^, ^38^, ^39^ One of the largest studies included 714 patients and found 53% of males and 28% of females with hypohidrosis.^30^ An association between the level of Vascular Endothelial Growth Factor A and sweating was also found.^36^ ERT did not improve hypohidrosis.^24^ Furthermore, the degree of hypohidrosis did not correlate with the frequency nor the severity of Fabry crises.^31^ Anhidrosis predicted the presence of significant renal disease.^23^ A significant difference in skin moisture between FD patients and healthy controls was found.^38^

A study of 38 female patients found that those with painful neuropathy had reduced sweat output, as measured by acetylcholine iontophoresis,^46^ compared to healthy controls. Moreover, sweat output significantly declined with age in patients but not in the control group.^46^

Nine studies reported lymphoedema (four cross‐sectional studies, three retrospective studies, two case series, *n* = 6487 patients).^22^, ^26^, ^27^, ^30^, ^34^, ^37^, ^40^, ^42^ It was the fourth most reported manifestation. Lymphoedema is a marker of greater systematic severity as assessed by the MSSI.^30^ A higher prevalence of lymphoedema in males was found in two studies.^37^, ^40^ One study found that the classic phenotype had the highest prevalence of lymphedema, occurring in 16.5% of 5487 patients assessed for lymphoedema.^40^ Among those with lymphedema, microlymphatic hypertension was also observed.^42^

Hair abnormalities were the least reported clinical manifestation of FD, with only two studies addressing the issue (two case series, *n* = 40 patients).^25^, ^43^ One study found no hair abnormalities in either males (*n* = 6) or females (*n* = 5).^25^ The other study reported slow hair growth, limited to the chin area, in a single patient.^43^ No studies discussed treatment regimens for restoring body hair density.

### 3.5 Risk of bias assessment and applicability

The QUADAS‐2 checklist showed a low risk of bias and applicability concerns in all domains in four studies.^24^, ^27^, ^36^, ^37^ The remaining studies had one to four items scored as “high” or “unclear risk and applicability concerns” (File S1).

## 4 DISCUSSION

A variety of skin manifestations in patients with FD are described, and most importantly the literature reports patients having angiokeratomas,^22^, ^23^, ^24^, ^25^, ^26^, ^27^, ^28^, ^29^, ^30^, ^31^, ^32^, ^33^, ^34^, ^35^, ^36^ telangiectasia,^22^, ^30^, ^32^, ^36^, ^37^ lymphoedema,^22^, ^26^, ^27^, ^30^, ^34^, ^37^, ^40^, ^41^, ^42^ sweat^22^, ^23^, ^24^, ^25^, ^26^, ^27^, ^29^, ^30^, ^31^, ^33^, ^36^, ^38^, ^39^ and hair abnormalities.^25^, ^43^

The 0.014% incidence rate among neonates suggests significant underrecognition of the disease in clinical practice, or an increased incidence of gene variants that may be of unknown significance or known not to be disease‐causing, highlighting the need to reevaluate clinical approaches, particularly in neonatal care.^47^ Many cases of the disease may go undiagnosed or misdiagnosed, leading to delayed or inadequate treatment and poor clinical outcomes. There is an urgent need to improve diagnostic protocols, such as routinely performing cascade screening once a proband is diagnosed.^1^ However, the inclusion of routine screening for neonates and others remains debated due to the potential identification of non‐disease‐causing variants.^48^

**FIGURE 3:**
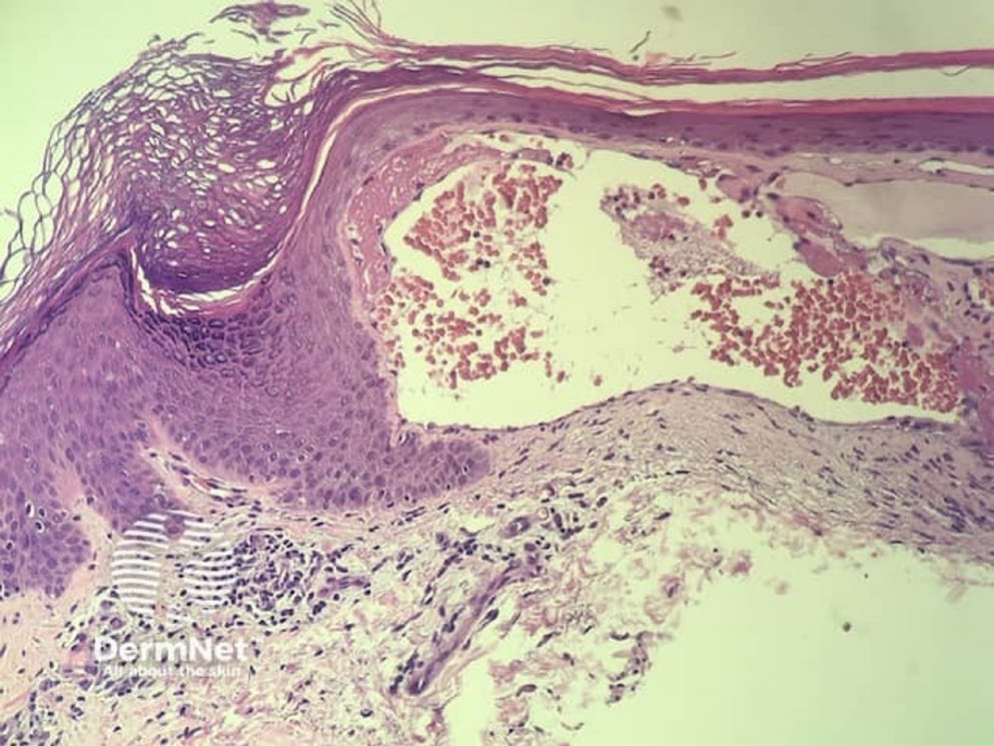
PRISMA flow diagram illustrating the literature search for skin manifestations in Fabry patients.

Extracutaneous manifestations are common and play a crucial role in facilitating early diagnosis, with prevalence rates ranging from 20% to 100%.^49^, ^50^, ^51^, ^52^, ^53^ Neuropathic pain, primarily in the hands and feet, results from glycolipid accumulation in small nerve fibers.^53^ Cornea verticillata, whorled corneal opacities detected through slit‐lamp examination, result from glycolipid deposition in corneal epithelial cells.^52^ Renal involvement (see Figure 3), leading to progressive insufficiency and end‐stage renal disease, stems from glycolipid buildup in glomeruli, tubules, and vascular endothelium.^51^ Mulberry cells are vacuolated epithelial cells resembling a mulberry filled with whirl‐shaped fat globules, known as mulberry bodies, found in urine sediment. Their origin remains unclear but linked to glycolipid buildup.^51^ Cardiac complications, such as left ventricular hypertrophy, arrhythmias, and valvular abnormalities, arise from glycolipid deposits in cardiac tissues.^50^ Gastrointestinal symptoms such as abdominal pain, diarrhea, and nausea are linked to glycolipid buildup in the autonomic nervous system and gastrointestinal smooth muscles.^49^

**FIGURE 4:**
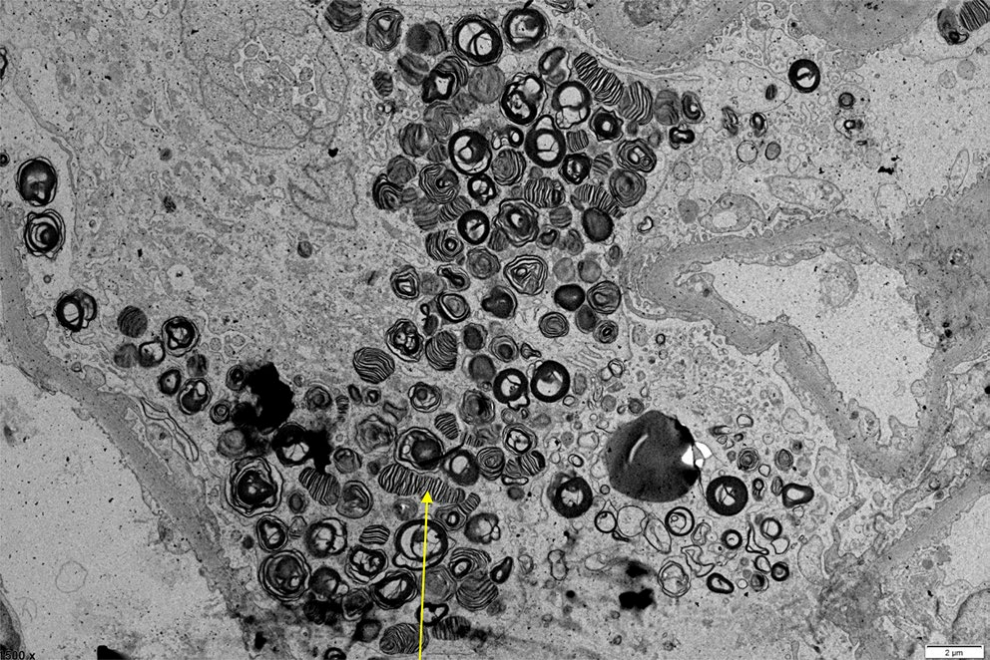
Histopathology image of an angiokeratoma. Image courtesy of DermNet, NZ, https://dermnetnz.org/topics/angiokeratoma‐pathology.

Apart from general supportive symptomatic treatment, the current disease‐specific treatment of FD is parenteral ERT, either Replagal, Fabrazyme or Elfabio and recently chaperone therapy Migalastat.^54^ ERT is administered every 2 weeks, targeting cells with glycosphingolipid accumulation. The enzyme helps to break down the accumulated substrate, alleviating symptoms, and limits or slows down tissue damage.^55^, ^56^ Some studies found ERT ineffective for skin manifestations,^24^, ^38^ while others suggest early initiation may improve long‐term outcomes.^35^, ^56^ Treatment has been available since 2001.^54^ Ongoing research is exploring the optimization of ERT dosing and administration to improve patient outcomes and reduce side effects. New formulations and delivery methods (e.g., every 4 weeks for the newly licensed ERT, Elfabrio®),^57^ along with gene therapy approaches, are being developed to improve ERT efficacy. However, challenges such as the cost, accessibility, need for lifelong, regular infusions, and development of antibodies against the enzyme are of significant concerns.^49^, ^54^, ^55^, ^58^, ^59^, ^60^, ^61^

In patients with amenable mutations, chaperone therapy works by stabilizing the patient's misfolded α‐galactosidase A enzyme, allowing it to reach the lysosomes and break down accumulated glycosphingolipids. Long‐term studies are ongoing to assess the durability of these effects. The oral administration of migalastat offers a significant advantage over intravenous ERT by improving patient convenience and treatment adherence.

The current literature is ambiguous. It is hypothesized that the progression of fibrosis in most organs cannot be reversed if a certain “point of no return” is reached, therefore most patients receiving ERT had lived with the disease for decades before initiating ERT. If the hypothesis about a “point of no return” wherein the emergence of disabling skin manifestations is assured in patients, clinicians should weigh the advantages of initiating early treatment.

Anderson et al. cited lymphoedema as a clinical sign of FD in the original description of the disorder.^62^ In patients with FD, lymphoedema appears to be related to the accumulation of glycosphingolipids in the lymphatic vessels rather than to the kidneys or because of cardiac disease.^62^ Lymphoedema is found in approximately 14% of patients with median age at onset of 49 and 39 years in females and males, respectively. In the absence of therapy, FD‐related lymphoedema may lead to erysipelas, making compression stockings mandatory.^62^

Møller et al.^39^ proposed that skin involvement in FD is age‐dependent and tends to increase with age, consistent with previous findings.^7^, ^10^ One study found that the onset of skin symptoms varies by phenotype, with late‐onset FD showing slower progression and milder symptoms compared to the classic variant.^63^, ^64^

The two largest cohorts found skin manifestations as a common feature in FD.^40^ In the Fabry Registry, lymphoedema occurred in 16.5% of 5487 patients assessed for lymphoedema with 84.5% receiving ERT, chaperone therapy or unknown types of treatment.^40^ Similarly, the Fabry Outcome Survey by Orteu et al. included 714 actively treated patients with relevant indications for ERT,^30^ chaperone therapy or other types of treatment, and thus already exhibited a certain degree of the disease prior to treatment, introducing some degree of bias.^30^

The rarity of FD has led to a limited body of research often based on small cohorts or low‐evidence case reports. The inclusion of studies with five or fewer participants and potential publication bias, favoring positive findings, may have introduced bias. Additionally, inconsistent and incomplete data on the extent, location, and severity of skin manifestations, combined with variability among studies, have made direct comparisons challenging and a meta‐analysis unfeasible.

The debilitating symptoms of FD demand a multidisciplinary evaluation. Diagnosis of FD is based on a disease‐causing variant in the *GLA* gene, reduced enzyme activity of α‐galactosidase A (blood test), and clinical findings. Skin lesions may continue to appear even under treatment, so a palliative measure should be offered when they impact the quality of life.^24^, ^38^, ^54^, ^65^ Treatment of skin lesions includes intense pulsed light and lasers, cryotherapy, electrocoagulation, or excisional surgery. Currently, the treatment of choice is laser therapy (neodymium YAG, pulsed dye, KTP 532 nm, copper, argon, candela V or beam laser).^66^, ^67^, ^68^

## 5 CONCLUSION

Sweat abnormalities were the most common skin manifestation, affecting 57.6% of patients. Angiokeratomas were observed in 51.5% of patients. Skin involvement seems age‐dependent and increases with age. The methodological quality of the studies was low and interstudy outcome variability was high. Skin changes were generally not reported to be downregulated by ERT. Several studies have indicated a correlation between skin manifestations and disease severity in FD. Inconsistent and incomplete data regarding the skin manifestations, combined with variability among studies, made direct comparisons challenging. Further research is needed to explore the pathogenesis, progression, and impact of FD on the skin.

## FUNDING INFORMATION

U.F.R.'s research salary was sponsored by a grant from the Kirsten and Freddy Johansen's Fund.

## CONFLICT OF INTEREST STATEMENT

U.F.R. received research grants, speaker and teaching honoraria, and travel costs for meetings from Genzyme, Freeline, Amicus and Protalix.

## CONSENT FOR PUBLICATION

All authors gave consent for publication.

## Supporting information


Data S1.

## Data Availability

All data are presented in the main manuscript.
