# Supplementary material for: Cutaneous manifestations of Fabry disease: A systematic review
Source: J Dermatol. 2025 Mar 7;52(4):571–82. doi: 10.1111/1346-8138.17690 (PMC11975216; doi:10.1111/1346-8138.17690)
Supplement: Supplementary file 1 — Data S1. [file JDE-52-571-s001.docx]

**Supplementary file 1**

Risk of bias and applicability assessment based on QUADAS-2 critical appraisal checklist.

| Study | RISK OF BIAS | | | | APPLICABILITY CONCERNS | | |
| --- | --- | --- | --- | --- | --- | --- | --- |
|  | **PATIENT SELECTION** | **INDEX TEST** | **REFERENCE STANDARD** | **FLOW AND TIMING** | **PATIENT SELECTION** | **INDEX TEST** | **REFERENCE STANDARD** |
| Alkhatib *et al.* 2023 | ☹ | ☺ | ☹ | ? | ☺ | ☺ | ☺ |
| Amann-Vesti *et al.* 2003 | ? | ? | ? | ☺ | ☺ | ? | ? |
| Anker *et al.* 2023 | ? | ☹ | ☹ | ☺ | ☺ | ☺ | ☺ |
| Eng *et al.* 2007 | ☺ | ☺ | ? | ☹ | ☺ | ☺ | ☺ |
| Galanos *et al.* 2002 | ? | ☺ | ? | ☺ | ☺ | ☺ | ? |
| Groot *et al.* 1968 | ? | ☺ | ? | ? | ☺ | ☺ | ? |
| Guinovart *et al.* 2013 | ☺ | ☺ | ☺ | ☺ | ☺ | ☺ | ☺ |
| Gupta *et al.* 2008 | ? | ☺ | ☺ | ☺ | ☺ | ☺ | ☺ |
| Larralde *et al.* 2004 | ? | ☺ | ☺ | ☺ | ☺ | ☺ | ☺ |
| Lidove *et al.* 2016 | ☺ | ☺ | ☺ | ☺ | ☺ | ☺ | ☺ |
| MacDermot *et al.* 2001 (a) | ☺ | ☺ | ☺ | ☺ | ☺ | ☺ | ☺ |
| MacDermot *et al.* 2001 (b) | ☹ | ☺ | ? | ☹ | ☺ | ☺ | ☹ |
| Wataya-Kaneda *et al.* 2023 | ? | ☺ | ☺ | ☺ | ☺ | ☺ | ☺ |
| Möhrenschlager *et al.* 2007 | ? | ☺ | ☺ | ☺ | ☺ | ☺ | ☺ |
| Møller *et al.* 2009 | ? | ☺ | ☹ | ? | ☺ | ☺ | ☺ |
| Nagai-Sangawa *et al.* 2021 | ? | ☺ | ☺ | ? | ☺ | ☺ | ☺ |
| Orteu *et al.* 2007 | ☺ | ☺ | ? | ☺ | ☺ | ☺ | ☺ |
| Spence *et al.* 1978 | ☹ | ☺ | ☺ | ☺ | ☺ | ☺ | ☺ |
| Wallace *et al.* 1965 | ☹ | ☹ | ? | ? | ☺ | ☺ | ? |
| Wattanasirichaigoon *et al.* 2006 | ☺ | ☺ | ? | ? | ☺ | ☺ | ? |
| Whybra *et al.* 2001 | ? | ☺ | ☺ | ☺ | ☺ | ☺ | ☺ |
| Wijburg *et al.* 2015 | ☺ | ☺ | ? | ☹ | ☺ | ☺ | ? |
| Zampetti *et al.* 2013 | ☺ | ☺ | ☺ | ☺ | ☺ | ☺ | ☺ |

☺Low Risk ☹High Risk ? Unclear Risk
